# Relationship between dynamic changes in remnant cholesterol and cardiovascular disease in middle-aged and older Chinese: a national cohort study

**DOI:** 10.3389/fcvm.2025.1503705

**Published:** 2025-06-19

**Authors:** Bin Zhang, Dengfeng Ma, Zhiqiang Pei, Qian Ren, Jin Qiu

**Affiliations:** ^1^ Department of Cardiology, Taiyuan City Central Hospital, Ninth Clinical Medical College of Shanxi Medical University, Taiyuan, China; ^2^ Oriental Pan-Vascular Devices Innovation College, University of Shanghai for Science and Technology, Shanghai, China; ^3^ Graduate School, Changzhi Medical College, Changzhi, Shanxi, China

**Keywords:** dynamic changes, remnant cholesterol, cardiovascular disease, middle-aged and older Chinese adults, national cohort study

## Abstract

**Aim:**

Epidemiological and genetic studies have shown that elevated basal remnant cholesterol (RC) levels increase cardiovascular disease (CVD) risk. However, the relationship between RC dynamics and CVD remains unclear. We aimed to investigate the relationship between the dynamic changes in RC and CVD occurrence in middle-aged and elderly populations in China.

**Methods and results:**

This cohort study investigated data from the study population of the 2011 and 2015 China Health and Retirement Longitudinal Study (CHARLS) and included 4,431 participants aged ≥45 years who provided complete information on RC and the occurrence or absence of CVD. Based on the change in RC in the population from 2011 to 2015, this study categorized the population into clusters 1, 2, and 3 using k-means cluster analysis. The relationship between baseline RC and CVD risk was examined using receiver operating characteristic (ROC) curves and restricted cubic spline (RCS) analysis. Logistic regression was used to explore the association between dynamic changes in RC levels and CVD risk, and subgroup analyses and sensitivity analyses were performed. There were 132 new cases of CVD among the 4,431 participants [2,386 women [53.85%] and 2,045 men [46.15%]; follow-up time, 4 years]. The area under the ROC curve for baseline RC in CVD was 0.534. RCS regression showed a linear association between RC at baseline and CVD. Logistic regression results showed a significantly increased CVD risk in Cluster 3 compared with Cluster 1 after correction for confounders (OR = 1.69, 95%CI: 1.13–2.55, *P* = 0.012); similarly, the risk of heart disease was significantly increased in Cluster 3 (OR = 1.76, 95%CI: 1.13–2.76, *P* = 0.012). Subgroup analyses showed a higher CVD risk in participants with baseline renal disease (OR = 15.34, 95%CI: 1.2–195.35, *P* = 0.035) and an interaction between RC change and body mass index (P for interaction = 0.038). Age-stratified analysis revealed a small difference in baseline RC between age groups (difference = −1.01 mg/dl, 95% CI: 0.08 to 1.94, *P* = 0.0328). RC showed the strongest correlation observed with TG (r = 0.80, *p* < 0.001).

**Conclusions:**

In middle-aged and older Chinese participants, increased dynamic RC predicts increased CVD risk. Therefore, continuous monitoring of changes in RC levels is needed to reduce the risk of CVD.

**Lay summary:**

This cohort study, including 4,431 middle-aged and elderly Chinese individuals, found that dynamically elevated remnant cholesterol was significantly associated with a 1.69-fold higher cardiovascular disease incidence, suggesting that dynamically elevated remnant cholesterol predicts a higher risk of cardiovascular disease in China's middle-aged and elderly population.

## Introduction

Cardiovascular diseases (CVD) are the most common cause of mortality worldwide (1). According to the 2022 Global Burden of Disease Report on Cardiovascular Disease, CVD will be responsible for an estimated 19.8 million deaths globally, with 396 million years of life lost and 44.9 million years lived with a disability (2). Over the last 30 years, the global age-standardized prevalence of CVD has remained almost unchanged. However, the residual risk of CVD is as high as 70% (3, 4). Compared to global statistics, the age-standardized prevalence of CVD in China is still continuously increasing, especially among the middle-aged and elderly populations, and the inflection point of the declining disease burden has not yet appeared (3). Therefore, further reduction in CVD residual risk while controlling for traditional risk factors is crucial for the early onset of the CVD prevalence inflection point.

Residual risks for CVD include lipid-associated lipoprotein(a), remnant cholesterol (RC), and apolipoprotein B, as well as non-lipid-associated inflammation, thrombosis, homocysteine, and uric acid (5–11). RC is gaining widespread attention as an emerging risk factor for atherosclerotic cardiovascular disease (ASCVD), with Wadström's (12) study finding that subjects with RC ≥ 1 mmol/L were associated with a twofold increase in mortality from cardiovascular and other diseases. RC [or triglyceride (TG), a commonly used marker of RC], also called triglyceride-rich lipoprotein cholesterol, consists of cholesterol in Very Low-Density Lipoproteins and Intermediate-Density Lipoproteins in the fasting state, as well as cholesterol in the chyme remaining after a meal (12). RC has mechanisms for developing atherosclerosis independent of Low-Density Lipoprotein Cholesterol (LDL-C). RC is more closely associated with arterial wall inflammation: for every 1 mmol/L increase in RC, C-reactive protein levels increase by 28% (13), whereas for LDL-C, they increase by only 7% (14). RC is more readily captured and taken up by macrophages, resulting in a faster rate of foam cell formation (15). Epidemiological and genetic studies have shown that elevated basal RC levels increase CVD risk (16, 17). However, the relationship between RC dynamics and CVD remains unclear.

Therefore, this study aimed to investigate the relationship between the dynamic changes in RC and CVD occurrence in middle-aged and elderly populations in China using the China Health and Retirement Longitudinal Study (CHARLS) database to further clarify the value of monitoring the changes in RC and to provide a reference for reducing CVD occurrence in this population.

## Methods

### Study population

This analysis was conducted using data from the CHARLS. CHARLS is a nationally representative longitudinal survey designed to investigate the social, economic, and health status of Chinese individuals aged ≥45 years (18). The baseline survey, Wave 1, was conducted from June 2011 to March 2012 and employed a multistage probability sampling method. Since then, CHARLS has released four follow-up waves of data: Waves 2, 3, 4, and 5 in 2013, 2015, 2018, and 2020, respectively.

**Figure 1:**
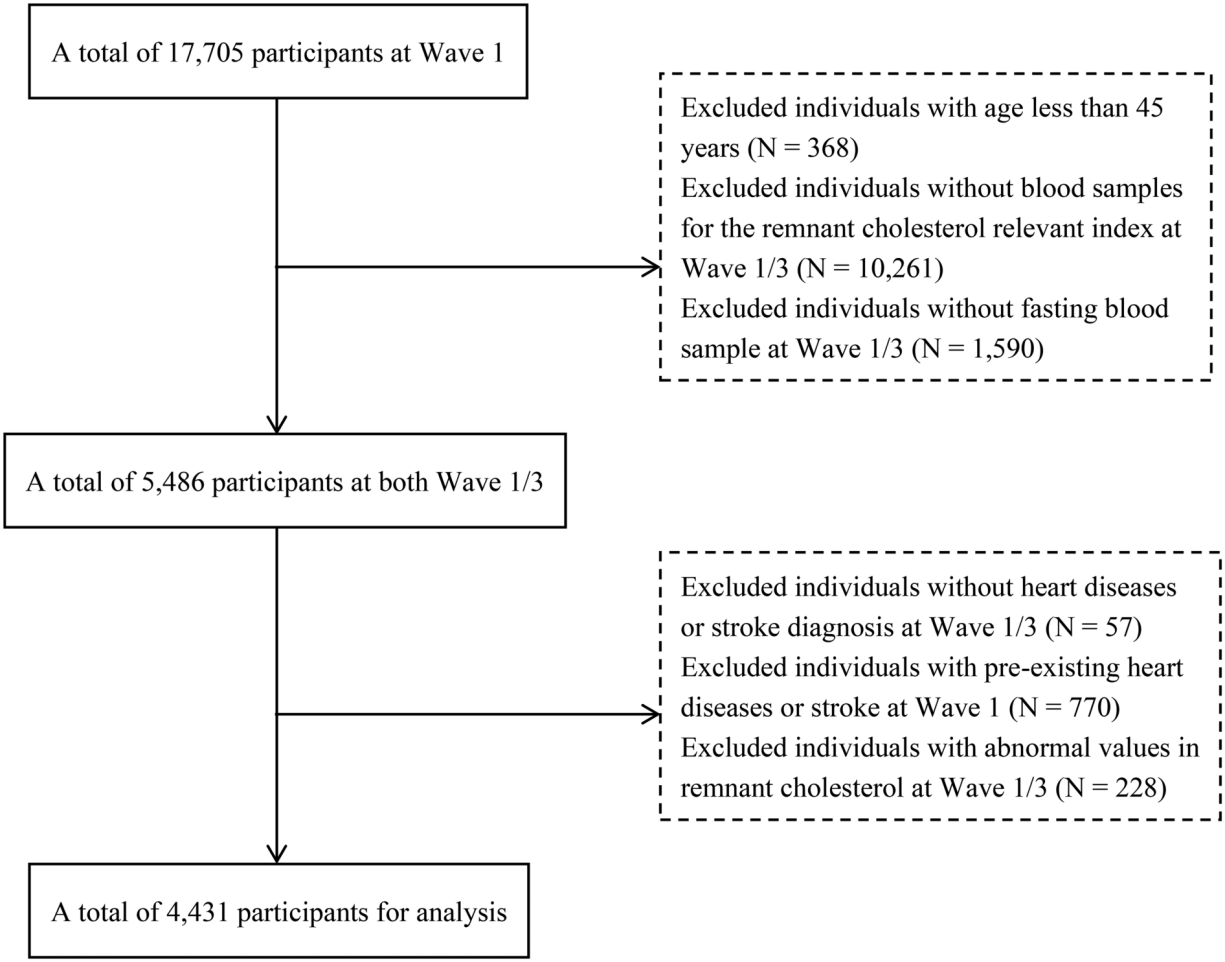
Flowchart of the study population from CHARLS.

Our analysis utilized follow-up data between Waves 1 and 3. Initially, the baseline survey in Wave 1 included 17,705 participants. Subsequently, we employed specific exclusion criteria (Figure 1) to select a final cohort of 4,431 participants. The exclusion criteria were as follows: (1) participants <45 years; (2) participants lacking RC-related blood sample indicators [Total Cholesterol(TC), High-Density Lipoprotein Cholesterol(HDL-C), and LDL-C] obtained in a fasting state; (3) participants without a diagnosis of heart disease or stroke in Waves 1 and 3; and (4) participants with a pre-existing condition of heart disease or stroke at baseline. (5) RC outliers in waves 1 and 3.

The Peking University Biomedical Ethics Review Board approved the data collection procedures for this study (IRB00001052-11015) and informed consent was obtained from all participants before they participated in the survey.

### Assessment of covariates

This study included baseline demographic variables and health-related indicators as confounding covariates. Demographic variables included age (“< 60” and “≥ 60” years), sex (“Male” and “Female”), hukou (“Agriculture” and “Others”), education level (“Primary school or lower” and “Secondary school or higher”), marriage status (“married” and “Divorced/Separated/Widowed/Never Married”). Health-related indicators included smoking status (“Yes,” “Quit,” and “No”), drinking status (“Yes,” “Quit” and “No”), BMI category (“Above Recommendations,” “Among Recommendations,” “Below Recommendations”). In the Chinese population, the optimal BMI range for participants aged 45–59 years is 18.5–23.9 kg/m^2^, while the optimal BMI range for participants aged ≥60 years is 22.0–26.9 kg/m^2^ (19). In addition, we considered whether participants had hypertension (self-reported or taking antihypertensive medication, or Systolic blood pressure ≥140 mmHg or Diastolic blood pressure ≥90 mmHg in the physical examination data (20), diabetes (self-reported or taking antidiabetic medication, or fasting plasma glucose ≥125 mg/dl or HbA1c ≥ 6.5% in the laboratory examination (21), and kidney disease (self-reported or taking medication for kidney disease).

For missing covariate values, we used the multiple interpolation method for imputation (22); the missing values and percentages for each variable are listed in Supplementary material Table S1.

### Definition of cardiovascular disease events

In this study, if a patient reported a diagnosis of heart disease (including myocardial infarction, coronary heart disease, angina, congestive heart failure, or other heart problems) or was diagnosed with stroke during the follow-up period, the participant was considered to have a positive result for a CVD event.

### Calculation of remnant cholesterol

We extracted TC, HDL-C, and LDL-C values from blood sample of waves 1 and 3 to calculate the RC values in those waves. The calculation was performed as TC (mg/dl) minus HDL-C(mg/dl) minus LDL-C(mg/dl) (23). The lipid panel was performed at the Youanmen Center for Clinical Laboratory of Capital Medical University. Their laboratory assays, coefficients of variation, and detection limits are indicated in the literature (24, 25).

We used the k-means clustering analysis results to investigate the changes in the participants’ RC during the follow-up process in waves 1 and 3.

### Statistical analysis

**Figure 2:**
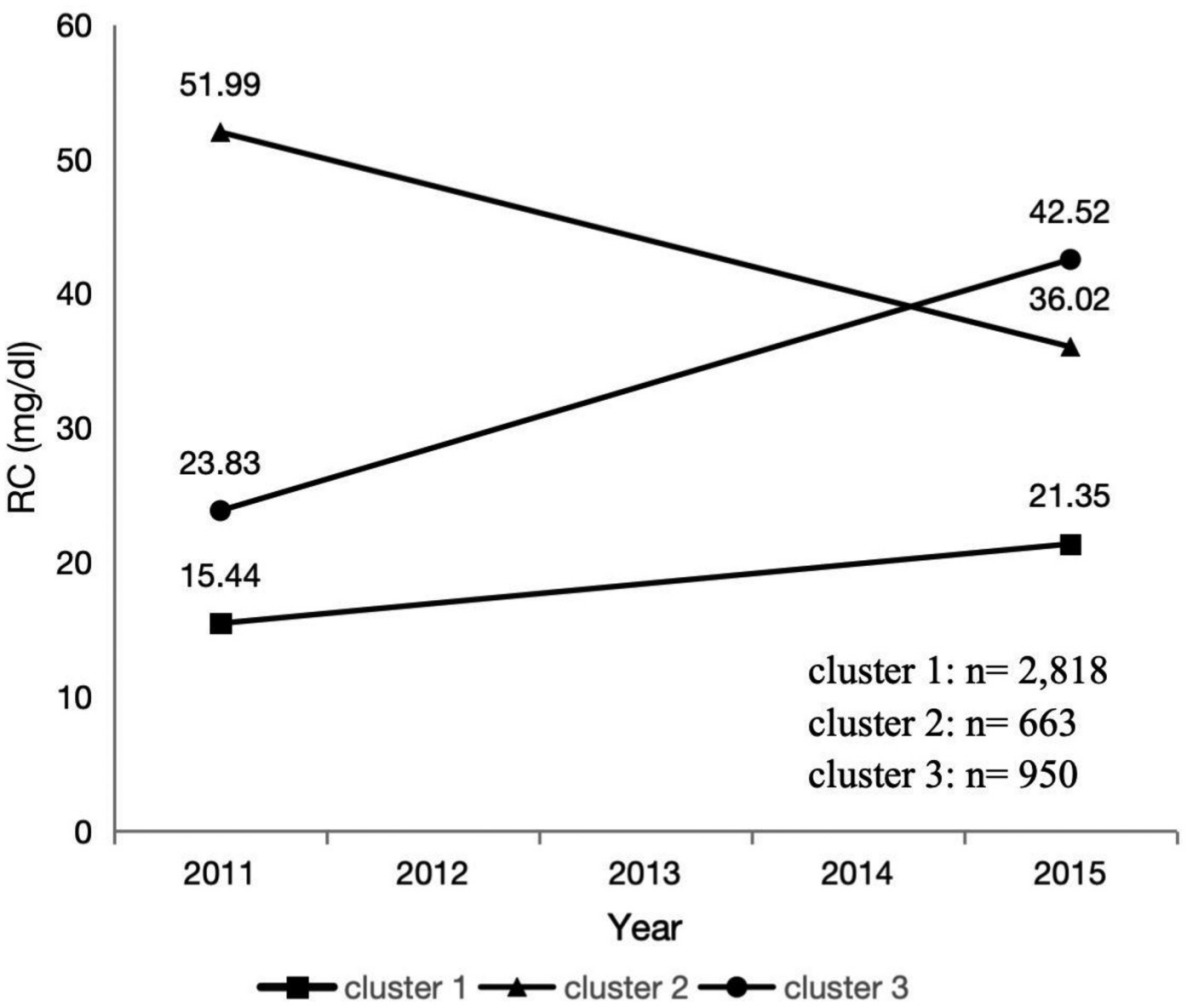
The RC clustering by k-means clustering.

We obtained the main trends in the RC between waves 1 and 3 using k-means clustering. K-means clustering is a rule-based method for determining the distances between data items. It is specifically described as “a method that partitions data into K clusters by iteratively relocating data points to their nearest cluster centroids, then updating centroids as the mean of assigned points, until convergence criteria (e.g., centroid stability or maximum iterations) are met.” The results showed that clustering analysis yielded the best results when the number of clusters was three (Supplementary material Figure S1). The three trends of change are shown in Figure 2: the first trend is considered as a low level and smooth change in RC from 15.44 mg/dl in 2011 to 21.35 mg/dl in 2015, the second trend is considered as a high level but intense decrease in RC from 51.99 mg/dl in 2011 to 36.02 mg/dl in 2015, and the third trend is considered as a low to high level and intense increase in RC from 23.83 mg/dl in 2011 to 42.52 mg/dl in 2015.

The participants’ baseline characteristics were described based on the k-means classification results. Categorical variables were represented by frequency and percentage, while continuous variables were described as mean [standard deviation (SD)] or median [interquartile range (IQR)], depending on their distribution. Based on the data type, we compared the differences between the different classification results using the Kruskal–Wallis test, chi-square test, and analysis of variance.

Diagnostic value analysis was performed using a receiver operating characteristic (ROC) curve, and the area under the curve was calculated to quantify the predictive ability of baseline RC for CVD. In addition, we explored the nonlinear relationship between baseline RC and CVD using restricted cubic spline (RCS) analysis with four nodes.

Subsequently, the association between changes in RC and CVD risk was examined using multivariable logistic regression. Four models were established for testing: the crude model without adjustment for any variables; Model 1 adjusted for age, sex, hukou, educational level, and marital status; Model 2 adjusted for the same variables as Model 1 plus smoking status, drinking status, and BMI category; and Model 3 further adjusted for hypertension, diabetes, and kidney diseases based on Model 2. A subgroup analysis was also conducted, which stratified the relationship between the above grouping variables and CVD and tested their interactions. To assess potential confounding factors, we performed two sensitivity analyses. First, we compared baseline RC levels between different age groups (<60 vs. ≥60 years) using t-tests. Second, we evaluated the relationship between RC and traditional lipid parameters (TC, HDL-C, LDL-C, and TG) using Spearman correlation analysis.

Similar, statistical analysis methods were used for heart disease and Stroke. Statistical analysis was performed using R 4.3.1. Statistical significance was set at *p* < 0.05.

## Results

### Baseline characteristics of participants

This study included 4,431 participants. Table 1 shows participants’ characteristics. Participants were predominantly <60 years old, with a higher percentage of females than males. Of the participants, 85.17% had an agricultural hukou, and 69.71% had attained an education level of primary school or lower. The proportion of married individuals, non-smokers, and non-drinkers was >50%. During the 4-year follow-up period, 132 new cases of CVD (2.98%) were identified, including 105 cases of heart disease (2.37%) and 29 cases of stroke (0.65%). The prevalence of baseline BMI above the recommended value was 44.34% and 42.32% in Clusters 2 and 3, respectively, compared to 24.52% in Cluster 1. Similarly, the prevalence of baseline hypertension was 46.91% and 44.11% in Clusters 2 and 3, respectively, compared to 36.16% in Cluster 1. The prevalence of diabetes at baseline was 23.08% and 17.58% in Clusters 2 and 3, respectively, compared to 10.22% in Cluster 1.

**Table 1:** Baseline characteristics of participants in renmant cholestreol analysis.

n	Level	Overall	Cluster 1	Cluster 2	Cluster 3	*p*
		*n* = 4,431	*n* = 2,818	*n* = 663	*n* = 950	
Age (%)	<60	2,522 (56.92)	1,548 (54.93)	383 (57.77)	591 (62.21)	0.0004
	≥60	1,909 (43.08)	1,270 (45.07)	280 (42.23)	359 (37.79)	
Sex (%)	Female	2,386 (53.85)	1,413 (50.14)	366 (55.20)	607 (63.89)	<0.0001
	Male	2,045 (46.15)	1,405 (49.86)	297 (44.80)	343 (36.11)	
Hukou (%)	Agriculture	3,774 (85.17)	2,435 (86.41)	539 (81.30)	800 (84.21)	0.0025
	Others	657 (14.83)	383 (13.59)	124 (18.70)	150 (15.79)	
Educational_level (%)	Primary school or lower	3,089 (69.71)	1,980 (70.26)	456 (68.78)	653 (68.74)	0.5752
	Secondary school or higher	1,342 (30.29)	838 (29.74)	207 (31.22)	297 (31.26)	
Marriage_status (%)	Divorced/seperated/widowed/never	438 (9.88)	292 (10.36)	55 (8.30)	91 (9.58)	0.2593
	Married	3,993 (90.12)	2,526 (89.64)	608 (91.70)	859 (90.42)	
Smoking_status (%)	No	2,758 (62.24)	1,699 (60.29)	416 (62.75)	643 (67.68)	0.001
	Quit	348 (7.85)	226 (8.02)	49 (7.39)	73 (7.68)	
	Yes	1,325 (29.90)	893 (31.69)	198 (29.86)	234 (24.63)	
Drinking_Status (%)	No	2,575 (58.11)	1,577 (55.96)	370 (55.81)	628 (66.11)	<0.0001
	Quit	346 (7.81)	227 (8.06)	58 (8.75)	61 (6.42)	
	Yes	1,510 (34.08)	1,014 (35.98)	235 (35.44)	261 (27.47)	
BMI (%)	Above recommendations	1,387 (31.30)	691 (24.52)	294 (44.34)	402 (42.32)	<0.0001
	Among recommendations	2,539 (57.30)	1,718 (60.97)	334 (50.38)	487 (51.26)	
	Below recommendations	505 (11.40)	409 (14.51)	35 (5.28)	61 (6.42)	
Hypertension (%)	No	2,682 (60.53)	1,799 (63.84)	352 (53.09)	531 (55.89)	<0.0001
	Yes	1,749 (39.47)	1,019 (36.16)	311 (46.91)	419 (44.11)	
Diabetes (%)	No	3,823 (86.28)	2,530 (89.78)	510 (76.92)	783 (82.42)	<0.0001
	Yes	608 (13.72)	288 (10.22)	153 (23.08)	167 (17.58)	
Kidney_diseases (%)	No	4,191 (94.58)	2,659 (94.36)	627 (94.57)	905 (95.26)	0.5663
	Yes	240 (5.42)	159 (5.64)	36 (5.43)	45 (4.74)	
FBG, mg/dl		108.039 (31.657)	104.767 (27.589)	116.287 (34.922)	111.985 (38.381)	<0.0001
TC, mg/dl		190.614 (34.703)	184.412 (33.013)	203.089 (35.694)	200.302 (34.364)	<0.0001
TG, mg/dl		118.305 (62.304)	90.001 (34.197)	220.804 (67.065)	130.732 (40.479)	<0.0001
HDL-C, mg/dl		50.933 (13.548)	54.498 (13.330)	40.644 (10.794)	47.541 (11.175)	<0.0001
LDL-C, mg/dl		114.820 [94.717, 136.856]	112.501 [92.784, 132.990]	111.341 [86.019, 132.410]	128.158 [107.958, 151.161]	<0.0001
HbA1c, mmol/mol		5.259 (0.775)	5.202 (0.674)	5.388 (0.832)	5.339 (0.974)	<0.0001
Remnant-C (2011), mg/dl		22.711 (15.717)	15.445 (8.298)	51.990 (12.965)	23.830 (8.631)	<0.0001
Remnant-C (2015), mg/dl		28.084 (12.490)	21.351 (5.839)	36.017 (13.848)	42.520 (10.298)	<0.0001
Cardiovascular disease (%)		132 (2.98)	69 (2.45)	22 (3.32)	41 (4.32)	0.0118
Heart disease (%)		105 (2.37)	56 (1.99)	14 (2.11)	35 (3.68)	0.0107
Stroke (%)		29 (0.65)	14 (0.50)	8 (1.21)	7 (0.74)	0.1133

BMI, body mass index; FBG, fasting blood glucose; TC, total cholesterol; TG, triglycerides; HDL-C, high-density lipoprotein cholesterol; LDL-C, low-density lipoprotein cholesterol; HbA1c, hemoglobin A1C; remnant-C, remnant cholesterol. Categorical variables are represented using frequencies and percentages, while normally distributed continuous variables are represented by means and standard deviations. Non-normally distributed continuous variables are represented using medians and interquartile ranges. To compare the differences between different classification results based on the data type, statistical tests such as the analysis of variance, chi-square test and Kruskal–Wallis test were employed.

### Restricted cubic splines analysis investigating the relationship between baseline RC and CVD

**Figure 3:**
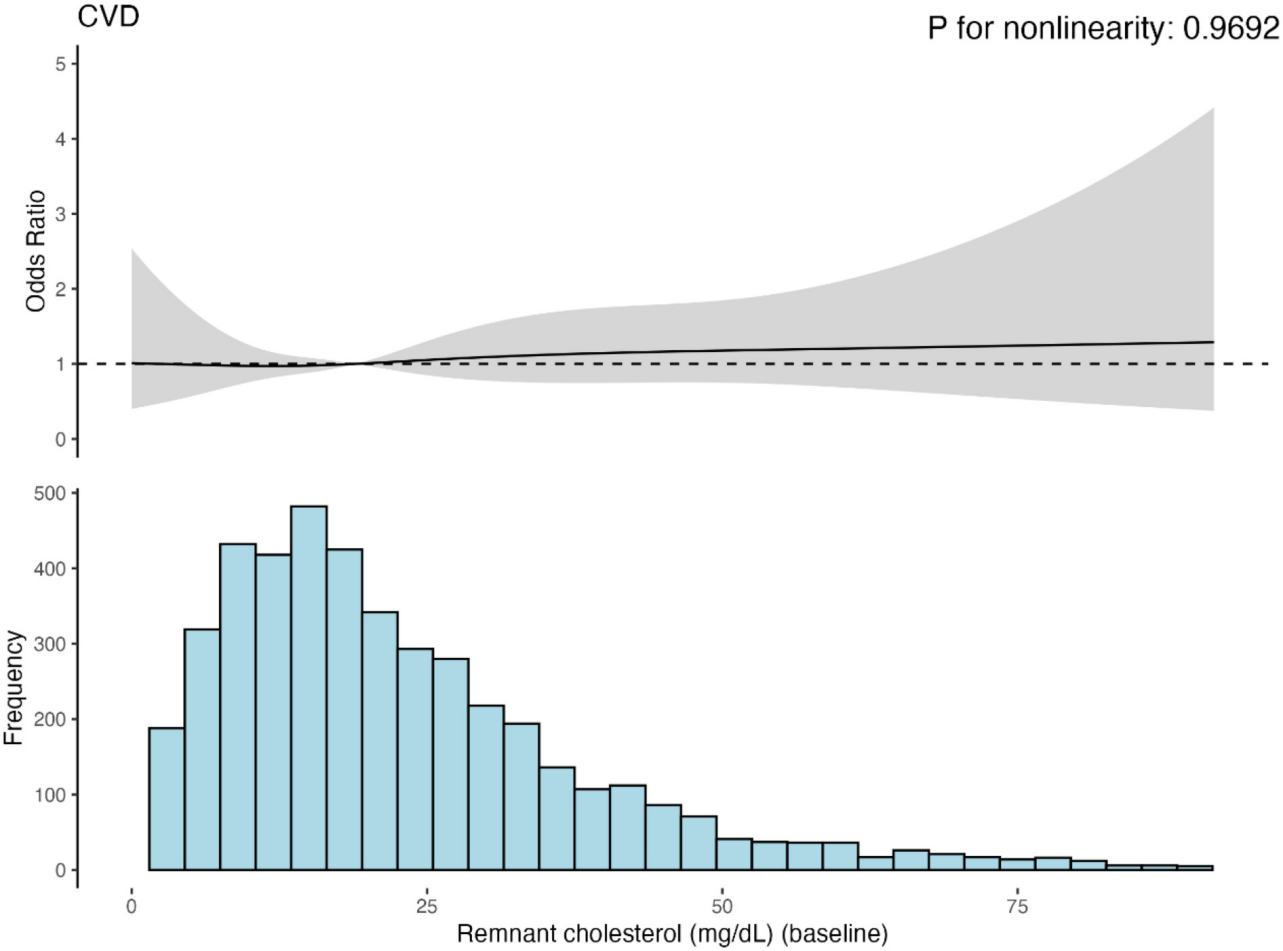
Cubic model of the relationship between baseling RC and CVD risk after adjusting for age, sex, hukou, educational level, marriage status, smoking status, dringking status, BMI category, hypertension, diabetes and kidney diseases.

Figure 3 presents the results of the RCS regression model. The relationship between baseline RC and CVD risk was linear. However, no statistically significant changes were observed in this trend. The relationships between baseline RC and heart disease or stroke risk were similar (Supplementary material Figures S5, S6). Besides, The ROC curve results of baseline RC and CVD, heart disease, or stroke, as shown in Supplementary material Figure S2–S4, also demonstrated some predictive power; however, the diagnostic performance was poor.

### Relationship between different clusters of RC and CVD

In logistic regression analysis, we investigated the association between different clusters and CVD (Table 2). Our results showed that, in the unadjusted model, cluster 3 exhibited a significant increase in CVD risk compared to the reference group (cluster 1) (OR = 1.80, 95%CI: 1.21–2.66, *P* = 0.004), while the correlation of cluster 2 was not significant (OR = 1.37, 95%CI: 0.84–2.23, *P* = 0.21). After adjusting for multiple confounding factors step-by-step, the association between Cluster 3 and CVD remained significant, and the risk ratio fluctuated across the models but showed a consistent overall trend, indicating that this association was relatively stable and not influenced by the considered confounding factors.

**Table 2:** Logistic regression analysis for the association between different classes and cardiovascular disease.

Cardiovascular Disease					
Groups	Cluster1	Cluster2		Cluster3	
		OR (95%CI)	*P*-value	OR (95%CI)	*P*-value
Crude	Ref	1.37 (0.84, 2.23)	0.21	1.80 (1.21, 2.66)	0.004
model 1	Ref	1.41 (0.86, 2.30)	0.17	1.89 (1.26, 1.81)	0.0002
model 2	Ref	1.33 (0.81, 2.19)	0.26	1.76 (1.17, 2.63)	0.0006
model 3	Ref	1.3 (0.78, 2.16)	0.32	1.69 (1.13, 2.55)	0.012

Model 1, adjusted for age, sex, hukou, educational level and marriage status. Model 2, adjusted for model 1, smoking status, drinking status and BMI. Model 3, adjusted for model 2, hypertension, diabetes and kidney diseases.

The association between different clusters and heart disease was similar to the above results, as shown in Supplementary material Table S2. The association analysis between different groups and stroke is worth mentioning (Supplementary material Table S3). In the Crude model, cluster 2 showed a significant positive correlation with stroke, with a risk ratio of 2.45 (95% CI: 1.02–5.86) and a *P* value of 0.046. In model 1, this association became even more significant (OR = 2.72, 95%CI: 1.13–6.56, *P* = 0.026). This appears to be contrary to our previous findings; however, after further adjustments, especially in model 3, this association did not reach statistical significance (OR = 2.2, 95%; CI: 0.87–5.57, *P* = 0.097).

### Subgroup analysis

**Figure 4:**
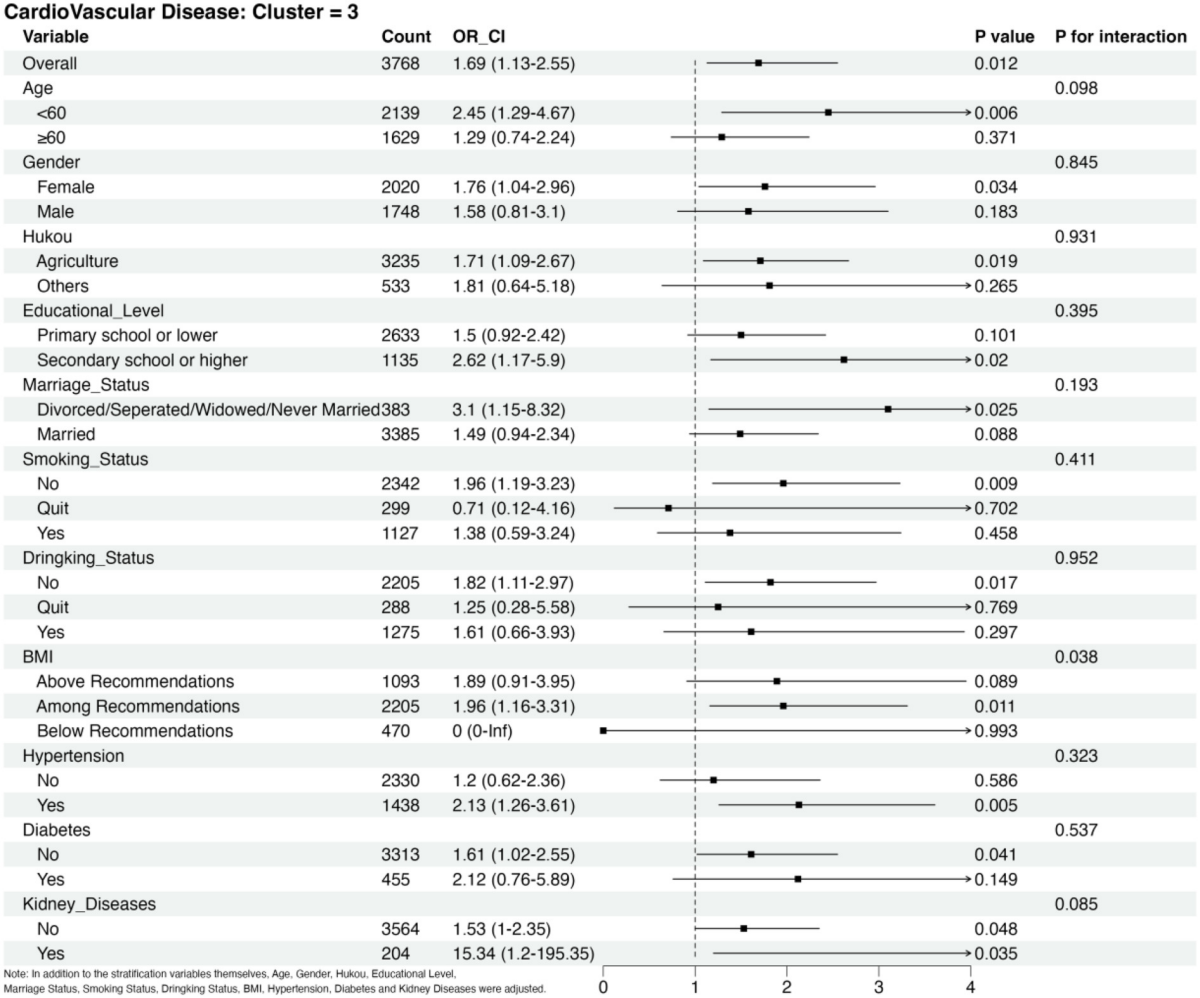
Subgroup analysis of CVD risks in cluster 3 adjusted for age, sex, hukou, educational level, marriage status, smoking status, drinking status, BMI category, hypertension, diabetes and kidney diseases in addition to the stratification variables themselves.

Figure 4 shows the findings of the subgroup analysis conducted for Cluster 3 using Cluster 1 as the reference group. Participants with baseline kidney disease had a higher CVD risk than other subgroups. In addition, we observed an interaction between changes in RC and BMI. No statistically significant results were observed in the analysis of the other subgroups, with no evidence of an interaction between the changes in RC and the subgroups (Supplementary material Figure S7–S11).

### Sensitivity analyses

Age-stratified analysis revealed a small difference in baseline RC between age groups (<60 vs. ≥60 years; difference = −1.01 mg/dl, 95% CI: 0.08–1.94, *P* = 0.0328) (Supplementary material Figure S12). The interaction between age and RC was not significant (P for interaction >0.05), suggesting consistent RC-CVD associations across age groups. RC showed significant correlations with TC (r = 0.18, *P* < 0.001), HDL-C (r = −0.47, *P* < 0.001), LDL-C (r = 0.048, *P* = 0.0016), and TG (r = 0.80, *P* < 0.001), with the strongest correlation observed with TG, suggesting the strong bivariate correlations (Supplementary material Figure S13).

## Discussion

The baseline RC level is strongly associated with CVD risk. This study further examined the relationship between dynamic changes in RC and CVD. This study of 4,431 individuals with up to 4 years of follow-up showed that a dynamic increase in RC was associated with a 1.69-fold increase in CVD risk in the population, and a decrease in RC levels was not independently associated with CVD.

The mechanisms through which elevated RC levels increase the risk of CVD have not yet been fully elucidated. Possible mechanisms are currently thought to include RC deposition in the arterial intima, macrophage activation, inflammation, and thrombus formation (26, 27). In this context, previous studies have shown that RC is a significant risk factor for ASCVD. When analyzed epidemiologically, the hazard ratios for myocardial infarction in patients with RC ≥ 1.5 mmol/L compared with RC < 0.5 mmol/L were 2.6 and 4.2, respectively [Copenhagen General Population Study (CGPS) and Copenhagen City Heart Study (CCHS)]; and the hazard ratios for ischemic stroke were 2 and 1.8, respectively (CGPS and CCHS) (16). A few dozens of single nucleotide polymorphisms associated with high RC levels were strongly and causally associated with adverse cardiovascular outcomes in 958,434 participants, and the effects on coronary heart disease and myocardial infarction were independent of LDL-C (17).

This study demonstrates in two ways that RC changes can influence CVD risk. In model 3 of the multivariate logistic regression analysis associated with CVD, the risk of CVD in cluster 3 was 1.69 times higher than cluster 1. In contrast, cluster 2, which had a high baseline RC level and strong decline, was not independently associated with the risk of CVD, suggesting that a decline in RC could reduce the association with CVD. Eicosapentaenoic Acid Ethyl Ester significantly reduced cardiovascular events in the previous REDUCE-IT study (28), a finding that may usher in a new era of cardiovascular therapy. However, studies related to RC and TG mainly were negative for the primary cardiovascular endpoints (29, 30). In conclusion, a dynamic decrease in RC is important for reducing the CVD risk.

RCS showed a linear relationship between changes in RC and CVD, suggesting that CVD risk increased with increasing RC levels; however, the trend was insignificant. Our findings are consistent with previous studies. A 2016 study by Joshi (31) found a linear association between RC levels and coronary heart disease events such as myocardial infarction, coronary heart disease death, and revascularization. Another study, which included two cohorts totaling over 100,000 persons, found a linear correlation between a higher RC and the risk of ischemic stroke (32).

In addition, interesting findings have emerged regarding the dynamics of RC and stroke. Although RC levels gradually decreased for Cluster 2 Model 2, the final risk ratio for this population was 2.51 times higher than that of Cluster 1, indicating that the RC level was still above the threshold for stroke risk factors. Previous studies have demonstrated that the critical RC concentration for CVD is 30 mg/dl (33). In this study, the ROC curve showed that the optimal critical concentration of RC was 26.68 mg/dl, which is similar to 30 mg/dl. However, the diagnostic efficacy for baseline RC in CVD was insufficient, probably due to differences in the study population. This is the first study to investigate the relationship between RC dynamics and CVD in the Chinese middle-aged and elderly population, and the ROC curve results may open a new way of thinking for this population, indicating the limitations of predicting CVD from a cross-section of RC levels in the Chinese middle-aged and elderly population, and also highlighting the study's significance.

Subgroup analyses showed that RC was a significant risk factor for CVD in the groups of hypertension, renal disease, no history of smoking or alcohol consumption, age <60 years, female sex, and unmarried status compared to Cluster 1. Many previous studies have shown that hypertension and renal disease are independent risk factors for developing CVD (3, 34); women <60 years of age account for approximately 50% of cases with no history of smoking or alcohol consumption. Extensive longitudinal studies of menopausal transition have shown changes in cardiovascular risk factors in women, including weight gain, visceral obesity, unfavorable changes in lipids, markers of inflammation, and increased blood pressure, which may be associated with an increased risk of ASCVD (35). Changes in marital status have also been associated with CVD morbidity and mortality. Results from a large-scale study published in 2022 based on over 620,000 individuals from the Asian Cohort Consortium showed that all-cause mortality was 15% higher in unmarried individuals and that the risk of death from all causes increased in those who were single, separated, divorced, or widowed (36). In addition, we observed an interaction between RC and BMI. Notably, BMI influences the effect of changing the RC on CVD. Tian (37) studied the correlation between RC and cardiovascular mortality in a Chinese population of 3.4 million people aged 46.2–66 years. The results showed that RC levels <22.0 mg/dl were more protective against CVD mortality in people with BMI < 24 kg/m² compared to those with BMI ≥ 24 kg/m². In contrast, those within the recommended BMI range had the highest risk of CVD. This may be due to the absence of CVD in the population below the recommended BMI, further influencing the modeling and interaction. This interaction could also be a false positive result.

Our study has several strengths. First, this is the first study to explore the association between dynamic changes in RC and the risk of CVD in middle-aged and elderly Chinese populations using cluster analysis. Cluster analysis classified the population into dynamically decreasing and dynamically increasing groups based on changes in RC and found that the incidence of cardiovascular events was significantly higher in the dynamically increasing group. In addition, this method avoids the limitation of being based on specific values. Second, we used data from a large-scale national longitudinal survey adjusted for multiple confounders, which could reflect the intrinsic association between dynamic changes in RC and new-onset CVD in Chinese middle-aged and elderly populations. In addition, RC, as a low-cost and convenient indicator, may be useful in predicting new-onset CVD in middle-aged and elderly Chinese populations.

This study has some limitations. First, due to the limited sample size, the sampling error was large, which may affect the accuracy and stability of the relationship between RC and new-onset CVD in the Chinese middle-aged and elderly population; second, the participants in this study were exclusively from China, and the results of this study cannot be directly extrapolated to other countries, and they need to be further confirmed by similar studies; third, CVD diagnosis was based on the internist report, and there was a lack of symptoms and gold standards for further evaluation; fourth, the exclusion of some patients without TC, HDL-C and LDL-C may have biased the conclusions; fifth, the study only had data from two blood samples, which did not allow for more detailed changes in RC; and sixth, the findings regarding stroke are questionable because of the limited number of stroke patients in this study, which may reduce the predictive efficacy for onset strokes.

## Conclusion

A middle-aged and older Chinese population with dynamically increasing RC was associated with a 1.69-fold greater risk of CVD; this relationship was particularly evident in participants with renal disease at baseline.

## Data Availability

The datasets presented in this study can be found in online repositories. The names of the repository/repositories and accession number(s) can be found below: https://charls.charlsdata.com/users/profile/index/zh-cn.html.
