# Supplementary material for: Relationship between dynamic changes in remnant cholesterol and cardiovascular disease in middle-aged and older Chinese: a national cohort study
Source: Front Cardiovasc Med. 2025 Jun 19;12:1503705. doi: 10.3389/fcvm.2025.1503705 (PMC12222158; doi:10.3389/fcvm.2025.1503705)
Supplement: Supplementary file 1 [file Datasheet1.pdf]

Table S1 Missing number for variables.

Variables	Missing number (%)
Age,years	7 (0.16%)
Sex(%)	1 (0.02%)
Hukou(%)	2 (0.05%)
Educational Level(%)	4 (0.09%)
Marriage Status(%)	31 (0.70%)
Smoking Status(%)	58 (1.31%)
Drinking Status(%)	7 (0.16%)
BMI, kg/m^2	585 (13.20%)
Hypertension(%)	21 (0.47%)
Diabetes(%)	34 (0.77%)
Kidney Diseases(%)	16 (0.36%)
GLU, mg/dl	5 (0.11%)
TC, mg/dl	0 (0)
TG, mg/dl	4 (0.09%)
HDL-C, mg/dl	0 (0)
LDL-C, mg/dl	0 (0)
HbA1c, mmol/mol	21 (0.47%)

Abbreviations: BMI: body mass index; GLU: glucose; TC: total cholesterol; TG: triglycerides; HDL-C: high-density lipoprotein cholesterol; LDL-C: low-density lipoprotein cholesterol; HbA1c: glycosylated hemoglobin

Table S2 Logistic regression analysis for the association between different classes and heart disease.

Heart Disease					
groups	cluster1	cluster2		cluster3	
		OR (95%CI)	P-value	OR (95%CI)	P-value
Crude	Ref	1.06(0.59,1.92)	0.84	1.89 (1.23, 2.90)	0.004
model 1	Ref	1.06(0.59,1.93)	0.84	1.93 (1.25, 2.98)	0.003
model 2	Ref	1.01(0.55,1.84)	0.97	1.81 (1.16, 2.81)	0.008
model 3	Ref	1.05(0.57,1.95)	0.872	1.76(1.13, 2.76)	0.012

Model 1, adjusted for age, sex, hukou, educational level and marriage status.

Model 2, adjusted for model 1, smoking status, drinking status and BMI.

Model 3, adjusted for model 2, hypertension, diabetes and kidney diseases.

Table S3 Logistic regression analysis for the association between different classes and stroke.

Stroke					
groups	cluster1	cluster2		cluster3	
		OR (95%CI)	P-value	OR (95%CI)	P-value
Crude	Ref	2.45 (1.02, 5.86)	0.046	1.49 (0.60, 3.69)	0.39
model 1	Ref	2.72 (1.13, 6.56)	0.026	1.77 (0.71, 4.46)	0.22
model 2	Ref	2.51 (1.02, 6.15)	0.04	1.59 (0.63, 4.04)	0.33
model 3	Ref	2.2 (0.87, 5.57)	0.097	1.49 (0.58, 3.82)	0.41

Model 1, adjusted for age, sex, hukou, educational level and marriage status.

Model 2, adjusted for model 1, smoking status, drinking status and BMI.

Model 3, adjusted for model 2, hypertension, diabetes and kidney diseases.

Figure S1 K-means clustering method for clustering the RC.

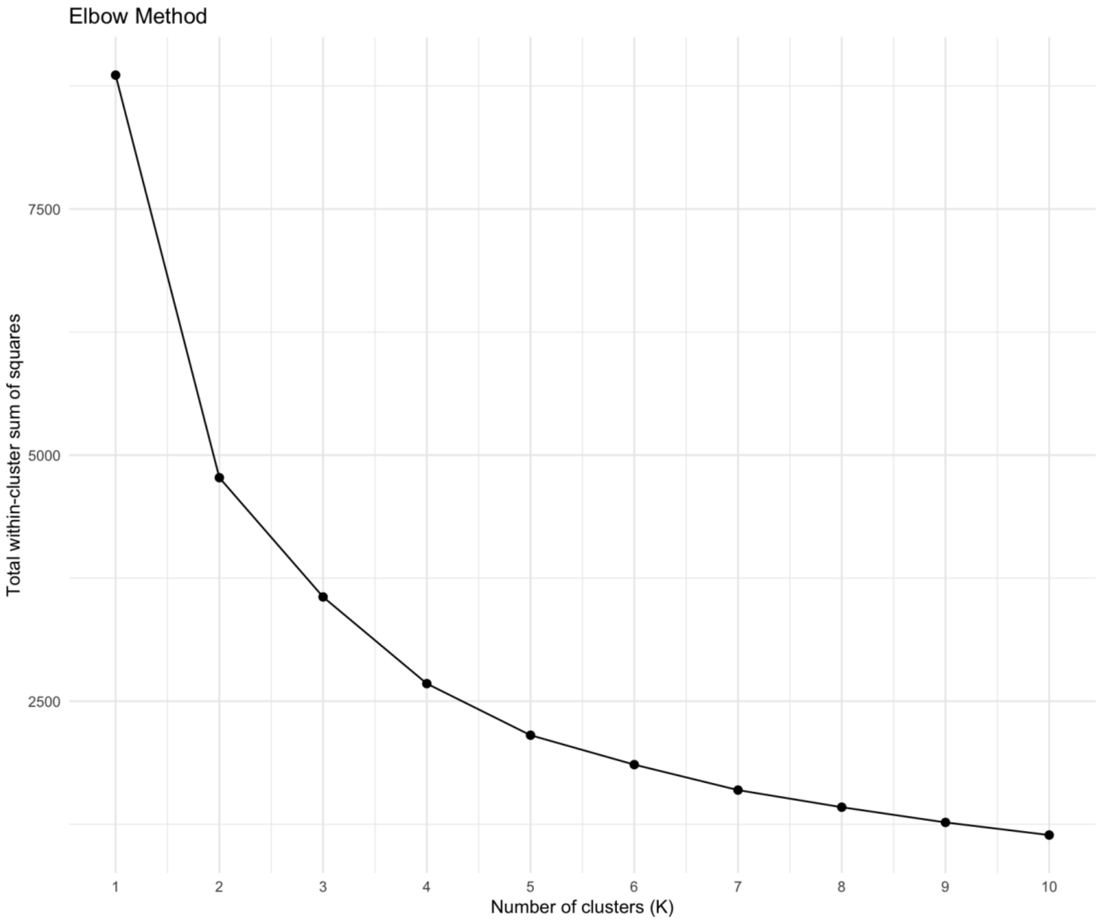

Figure S2 ROC curves of baseline RC in relation to CVD.

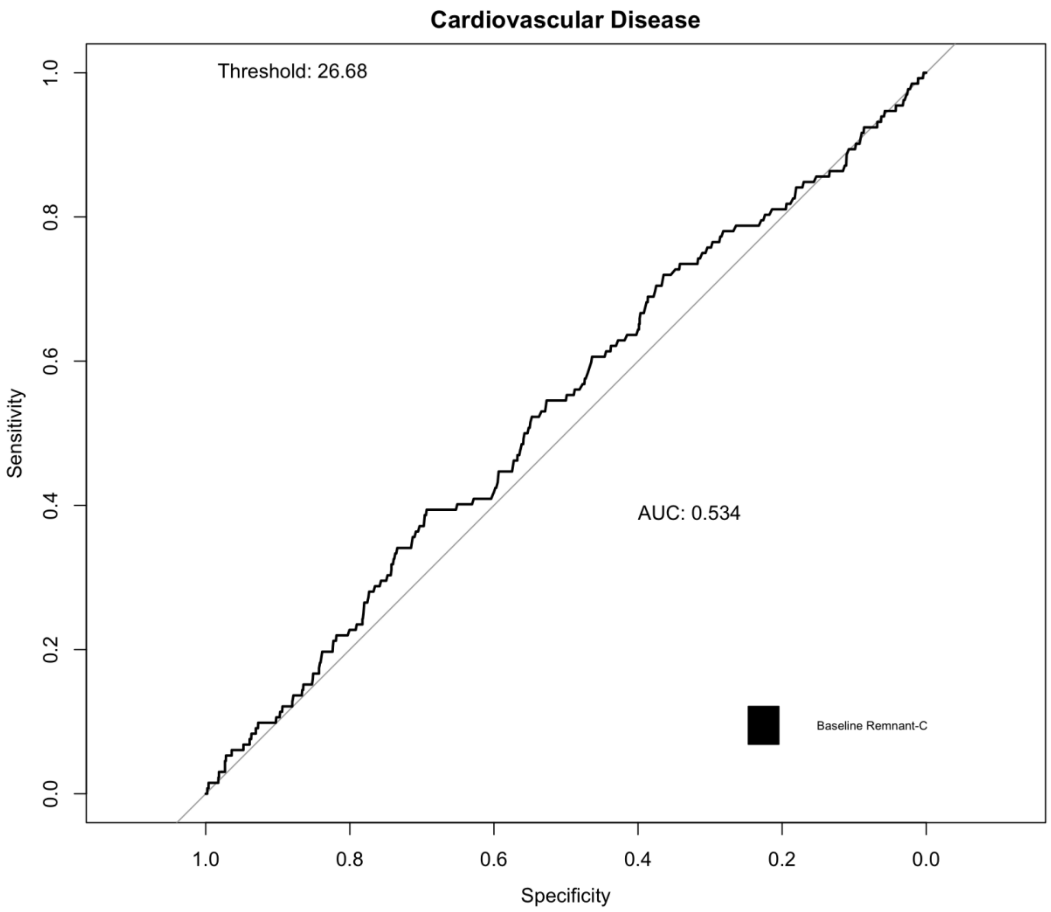

Figure S3 ROC curves of baseline RC in relation to Heart Disease.

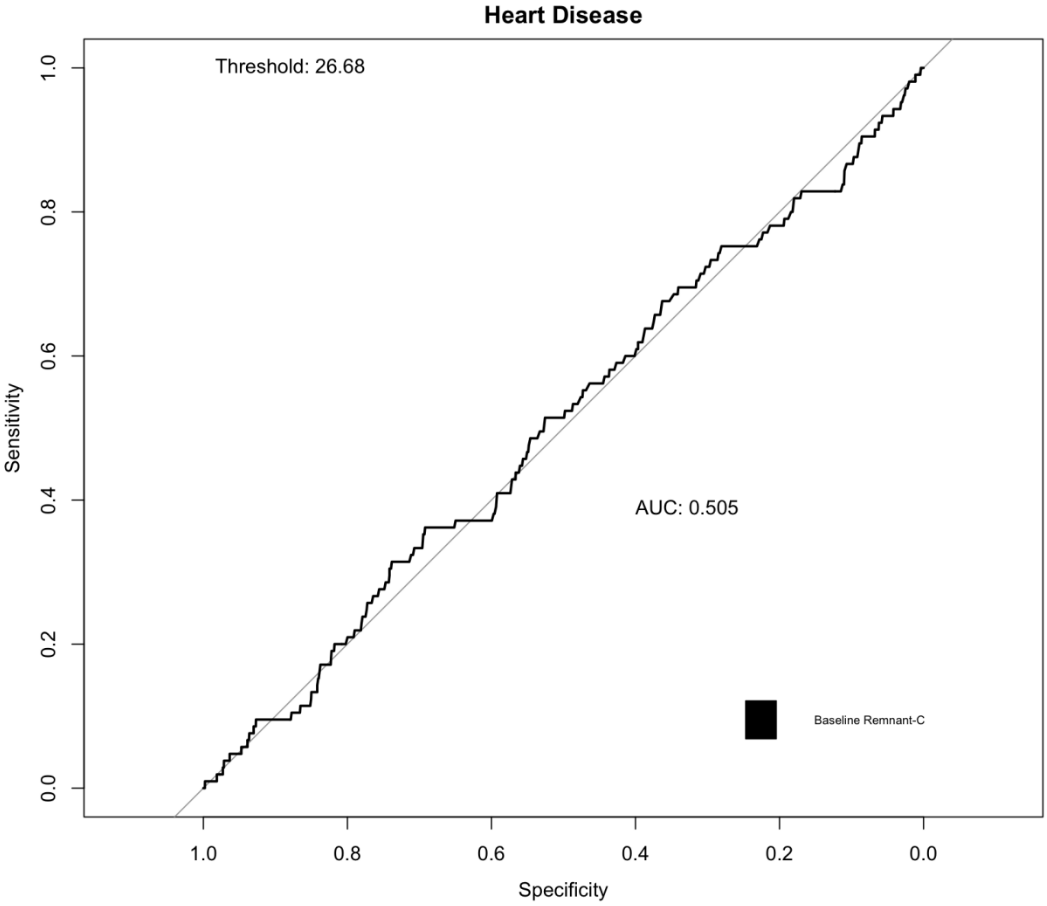

Figure S4 ROC curves of baseline RC in relation to Stroke.

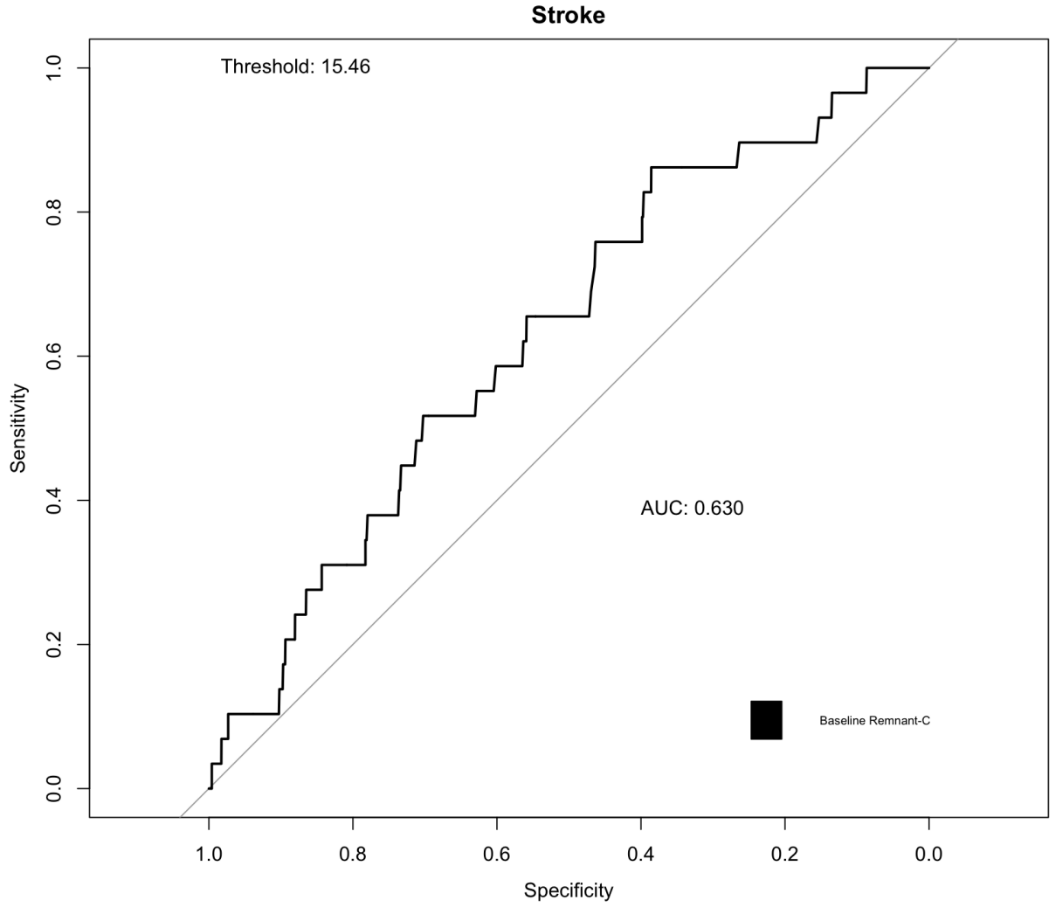

Figure S5 Cubic model of the relationship between baseling RC and heart disease risk after adjusting for age, sex, hukou, educational level, marriage status, smoking status, drinking status, BMI category, hypertension, diabetes and kidney diseases.

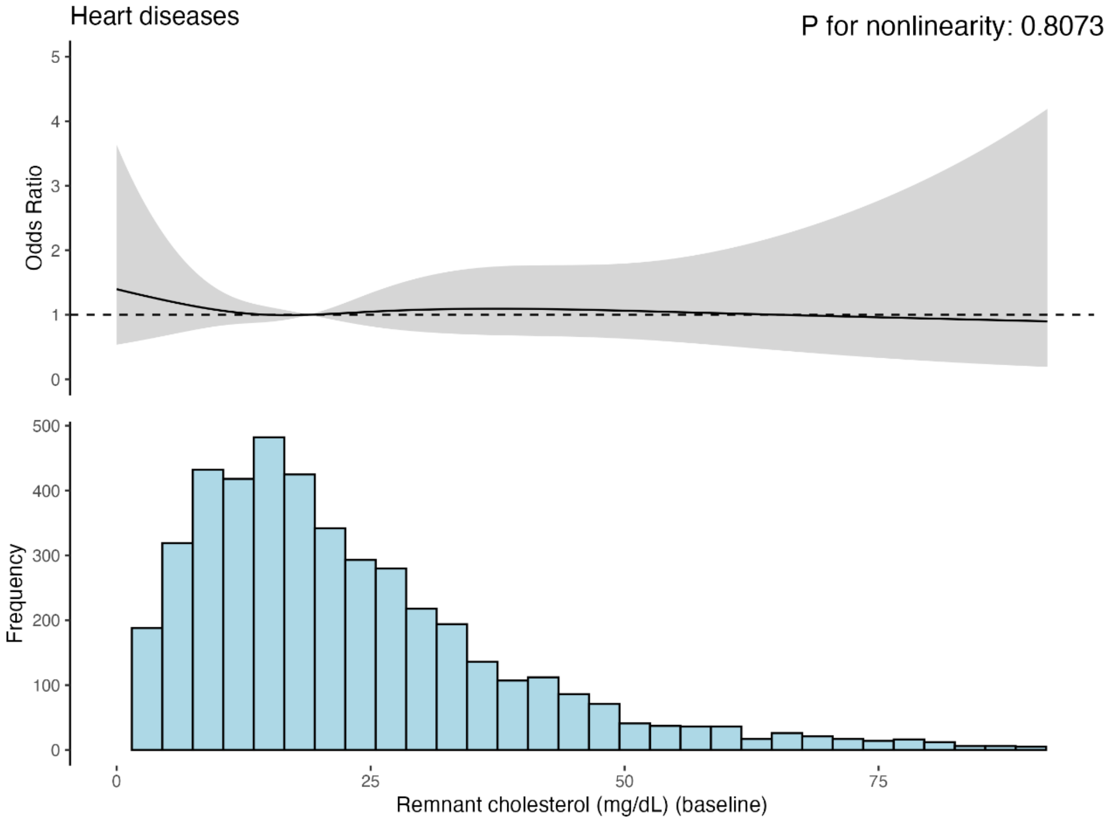

Figure S6 Cubic model of the relationship between baseling RC and stroke risk after adjusting for age, sex, hukou, educational level, marriage status, smoking status, drinking status, BMI category, hypertension, diabetes and kidney diseases.

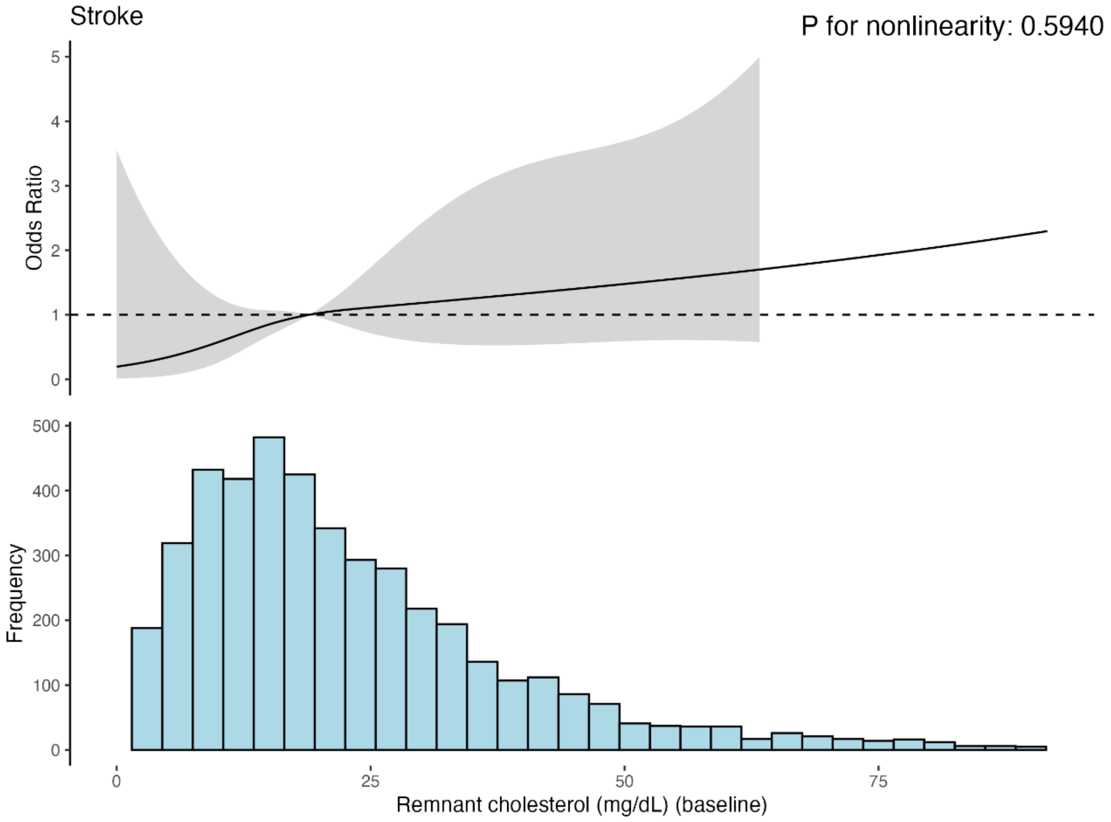

Figure S7 Subgroup analysis of CVD risks in cluster 2 adjusted for age, sex, hukou, educational level, marriage status, smoking status, drinking status, BMI category, hypertension, diabetes and kidney diseases in addition to the stratification variables themselves.

CardioVascular Disease: Cluster = 2

Variable	Count	OR_CI		P value	P for interaction
Overall	3481	1.3 (0.78-2.16)		0.318	
Age					0.232
<60	1931	1.97 (0.88-4.39)		0.099	
≥60	1550	1.06 (0.54-2.09)		0.869	
Gender					0.566
Female	1779	1.25 (0.63-2.51)		0.523	
Male	1702	1.54 (0.72-3.32)		0.267	
Hukou					0.483
Agriculture	2974	1.42 (0.82-2.47)		0.21	
Others	507	0.8 (0.2-3.27)		0.757	
Educational_Level					0.613
Primary school or lower	2436	1.37 (0.78-2.43)		0.276	
Secondary school or higher	1045	1.21 (0.37-3.99)		0.749	
Marriage_Status					0.33
Divorced/Seperated/Widowed/Never Married	347	3.34 (0.89-12.56)		0.075	
Married	3134	1.18 (0.67-2.08)		0.558	
Smoking_Status					0.716
No	2115	1.46 (0.78-2.75)		0.241	
Quit	275	1.73 (0.34-8.85)		0.513	
Yes	1091	0.88 (0.28-2.73)		0.82	
Dringking_Status					0.33
No	1947	1.64 (0.89-3.01)		0.11	
Quit	285	0.36 (0.04-3.3)		0.366	
Yes	1249	1.16 (0.37-3.61)		0.794	
BMI					0.234
Above Recommendations	985	1.67 (0.72-3.87)		0.231	
Among Recommendations	2052	1.35 (0.68-2.67)		0.384	
Below Recommendations	444	0 (0-Inf)		0.995	
Hypertension					0.714
No	2151	1.34 (0.62-2.92)		0.46	
Yes	1330	1.33 (0.67-2.64)		0.413	
Diabetes					0.918
No	3040	1.33 (0.74-2.37)		0.34	
Yes	441	1.28 (0.42-3.95)		0.663	
Kidney_Diseases					0.728
No	3286	1.3 (0.77-2.19)		0.325	
Yes	195	140543409.71 (0-Inf)		0.997	

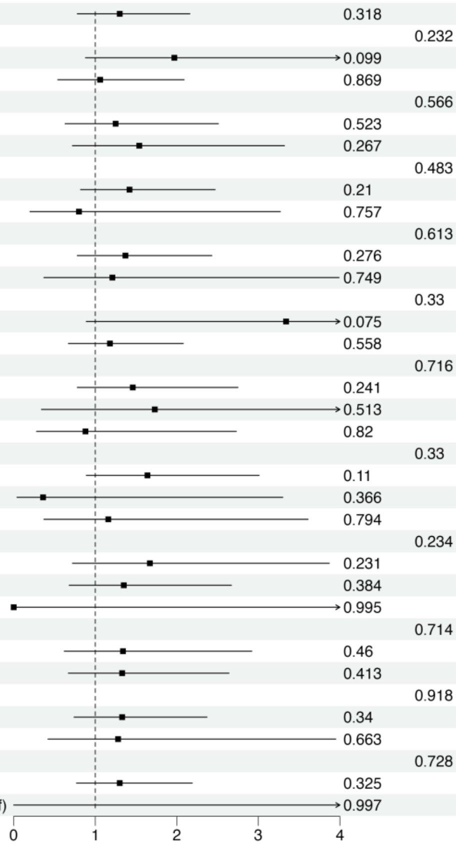

Note: In addition to the stratification variables themselves, Age, Gender, Hukou, Educational Level, Marriage Status, Smoking Status, Dringking Status, BMI, Hypertension, Diabetes and Kidney Diseases were adjusted.

Figure S8 Subgroup analysis of heart disease risks in cluster 2 adjusted for age, sex, hukou, educational level, marriage status, smoking status, drinking status, BMI category, hypertension, diabetes and kidney diseases in addition to the stratification variables themselves.

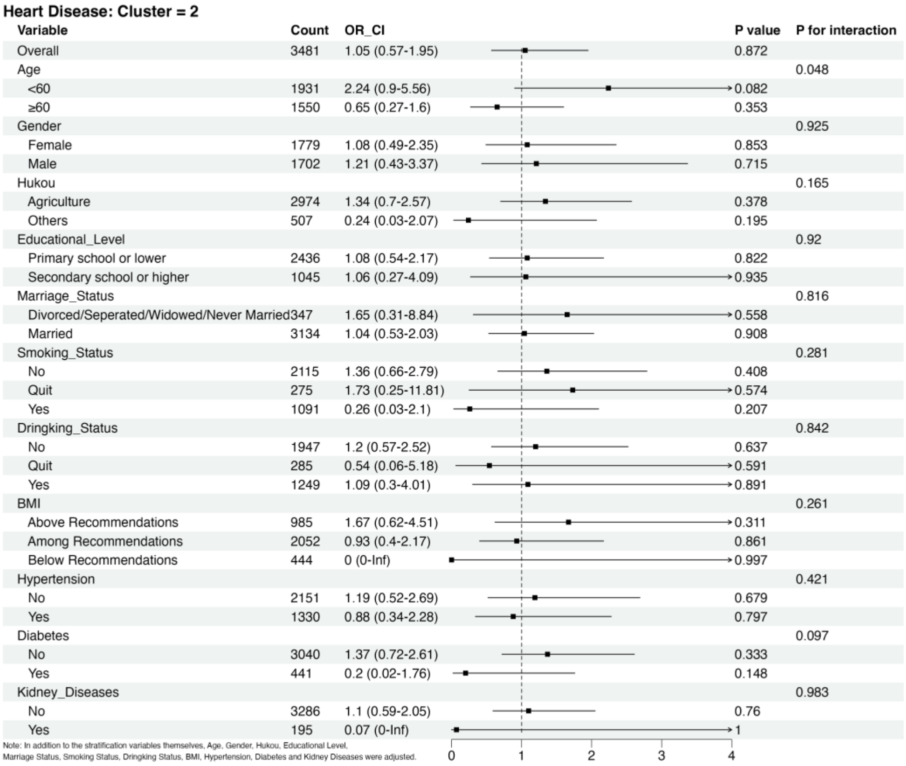

Figure S9 Subgroup analysis of heart disease risks in cluster 3 adjusted for age, sex, hukou, educational level, marriage status, smoking status, drinking status, BMI category, hypertension, diabetes and kidney diseases in addition to the stratification variables themselves.

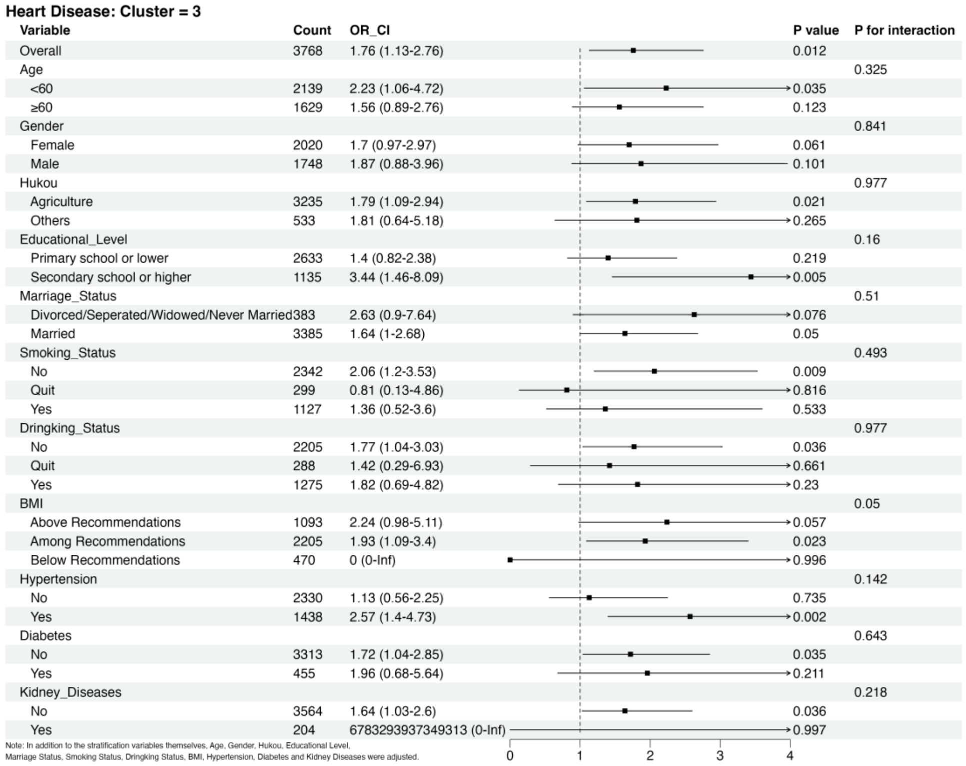

Heart Disease: Cluster = 3

Variable	Count	OR_CI	P value	P for interaction
Overall	3768	1.76 (1.13-2.76)	0.012	
Age				0.325
<60	2139	2.23 (1.06-4.72)	0.035	
≥60	1629	1.56 (0.89-2.76)	0.123	
Gender				0.841
Female	2020	1.7 (0.97-2.97)	0.061	
Male	1748	1.87 (0.88-3.96)	0.101	
Hukou				0.977
Agriculture	3235	1.79 (1.09-2.94)	0.021	
Others	533	1.81 (0.64-5.18)	0.265	
Educational_Level				0.16
Primary school or lower	2633	1.4 (0.82-2.38)	0.219	
Secondary school or higher	1135	3.44 (1.46-8.09)	0.005	
Marriage_Status				0.51
Divorced/Seperated/Widowed/Never Married	383	2.63 (0.9-7.64)	0.076	
Married	3385	1.64 (1-2.68)	0.05	
Smoking_Status				0.493
No	2342	2.06 (1.2-3.53)	0.009	
Quit	299	0.81 (0.13-4.86)	0.816	
Yes	1127	1.36 (0.52-3.6)	0.533	
Dringking_Status				0.977
No	2205	1.77 (1.04-3.03)	0.036	
Quit	288	1.42 (0.29-6.93)	0.661	
Yes	1275	1.82 (0.69-4.82)	0.23	
BMI				0.05
Above Recommendations	1093	2.24 (0.98-5.11)	0.057	
Among Recommendations	2205	1.93 (1.09-3.4)	0.023	
Below Recommendations	470	0 (0-Inf)	0.996	
Hypertension				0.142
No	2330	1.13 (0.56-2.25)	0.735	
Yes	1438	2.57 (1.4-4.73)	0.002	
Diabetes				0.643
No	3313	1.72 (1.04-2.85)	0.035	
Yes	455	1.96 (0.68-5.64)	0.211	
Kidney_Diseases				0.218
No	3564	1.64 (1.03-2.6)	0.036	
Yes	204	6783293937349313 (0-Inf)	0.997	

Note: In addition to the stratification variables themselves, Age, Gender, Hukou, Educational Level, Marriage Status, Smoking Status, Dringking Status, BMI, Hypertension, Diabetes and Kidney Diseases were adjusted.

Figure S10 Subgroup analysis of stroke risks in cluster 2 adjusted for age, sex, hukou, educational level, marriage status, smoking status, drinking status, BMI category, hypertension, diabetes and kidney diseases in addition to the stratification variables themselves.

Stroke: Cluster = 2

Variable	Count	OR_CI	P value	P for interaction
Overall	3481	2.2 (0.87-5.57)	0.097	
Age				0.444
<60	1931	1.39 (0.24-7.99)	0.714	
≥60	1550	2.62 (0.84-8.14)	0.096	
Gender				0.92
Female	1779	2.78 (0.52-15)	0.234	
Male	1702	2.09 (0.66-6.62)	0.208	
Hukou				0.985
Agriculture	2974	1.67 (0.61-4.61)	0.321	
Others	507	2.94460217989312e+25 (0-Inf)	0.998	
Educational_Level				0.463
Primary school or lower	2436	2.49 (0.89-6.93)	0.081	
Secondary school or higher	1045	1.87 (0.16-21.38)	0.613	
Marriage_Status				0.159
Divorced/Seperated/Widowed/Never Married	347	1.7698330397008e+25 (0-Inf)	0.997	
Married	3134	1.65 (0.59-4.65)	0.34	
Smoking_Status				0.951
No	2115	2.13 (0.54-8.32)	0.279	
Quit	275	0.71 (0.03-19.8)	0.841	
Yes	1091	3.02 (0.64-14.25)	0.162	
Dringking_Status				0.114
No	1947	4 (1.25-12.79)	0.019	
Quit	285	0 (0-Inf)	0.999	
Yes	1249	1.56 (0.17-14.49)	0.698	
BMI				0.693
Above Recommendations	985	1.75 (0.34-9.06)	0.506	
Among Recommendations	2052	3.38 (1.02-11.21)	0.046	
Below Recommendations	444	0 (0-Inf)	0.999	
Hypertension				0.507
No	2151	75.73 (0.85-6746.64)	0.059	
Yes	1330	2.09 (0.78-5.6)	0.145	
Diabetes				0.072
No	3040	1.1 (0.3-4.01)	0.886	
Yes	441	63.42 (2.03-1980.26)	0.018	
Kidney_Diseases				0.984
No	3286	2 (0.76-5.29)	0.16	
Yes	195	1263933402.19 (0-Inf)	1	

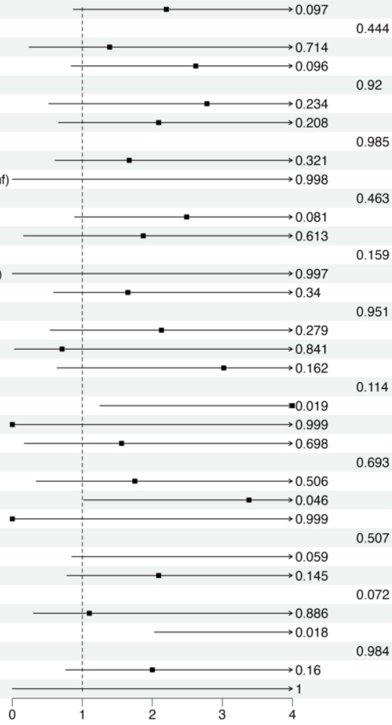

Note: In addition to the stratification variables themselves, Age, Gender, Hukou, Educational Level, Marriage Status, Smoking Status, Dringking Status, BMI, Hypertension, Diabetes and Kidney Diseases were adjusted.

Figure S11 Subgroup analysis of stroke risks in cluster 3 adjusted for age, sex, hukou, educational level, marriage status, smoking status, drinking status, BMI category, hypertension, diabetes and kidney diseases in addition to the stratification variables themselves.

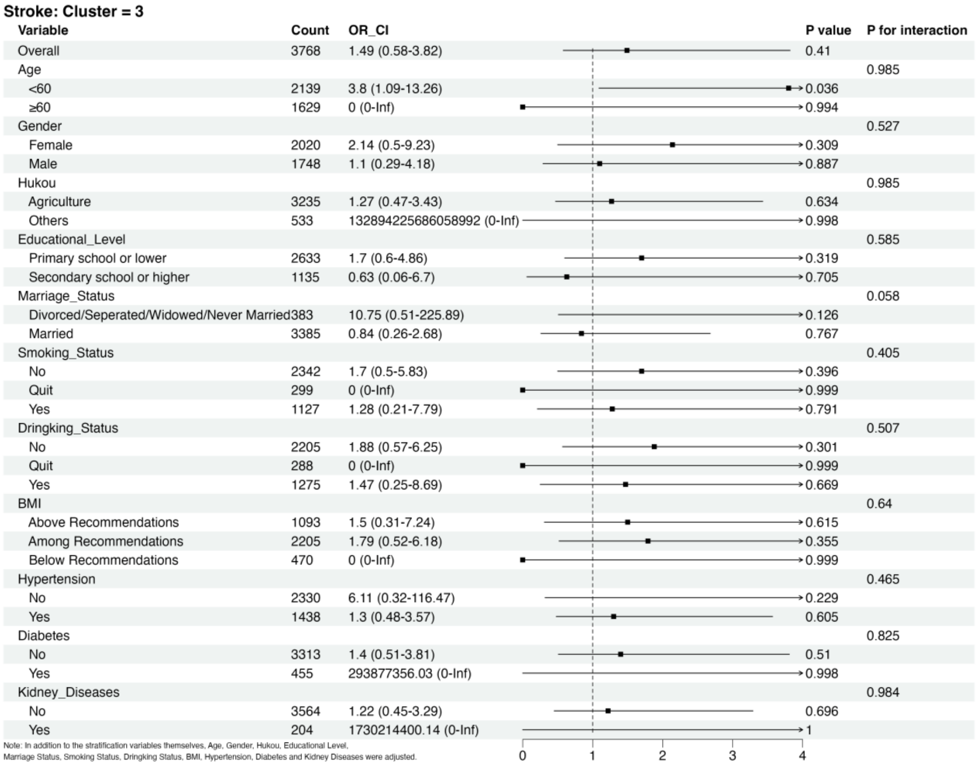

Figure S12 Relationship between baseline RC and different age groups.

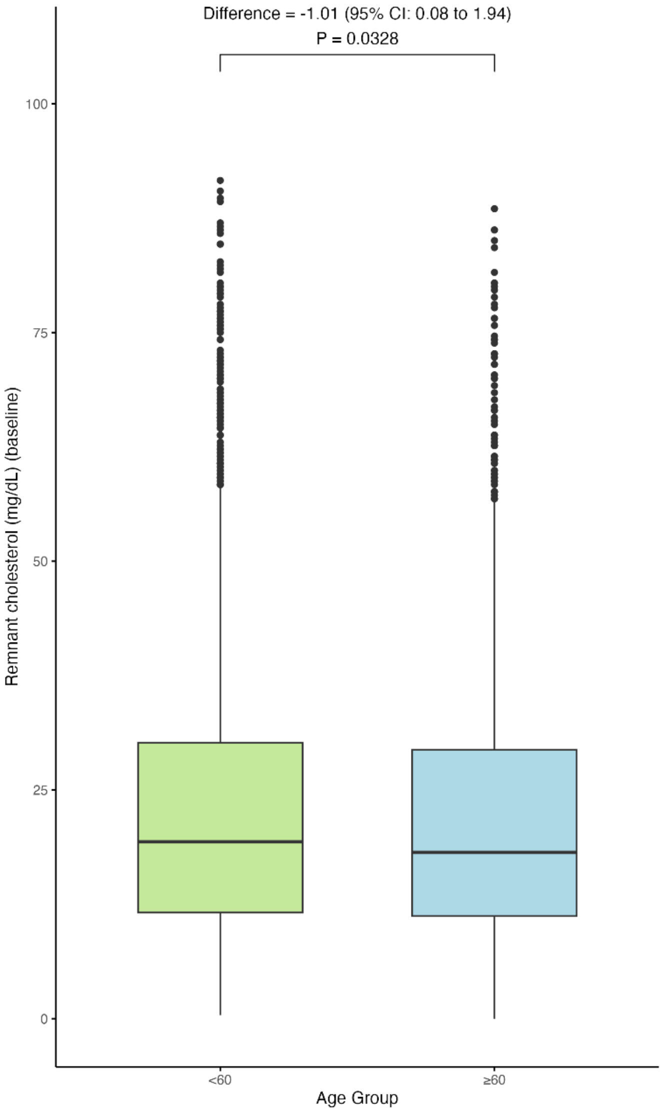

Figure S13 Spearman correlation analysis of RC and traditional lipid parameters (TC, HDL-C, LDL-C, and TG) .

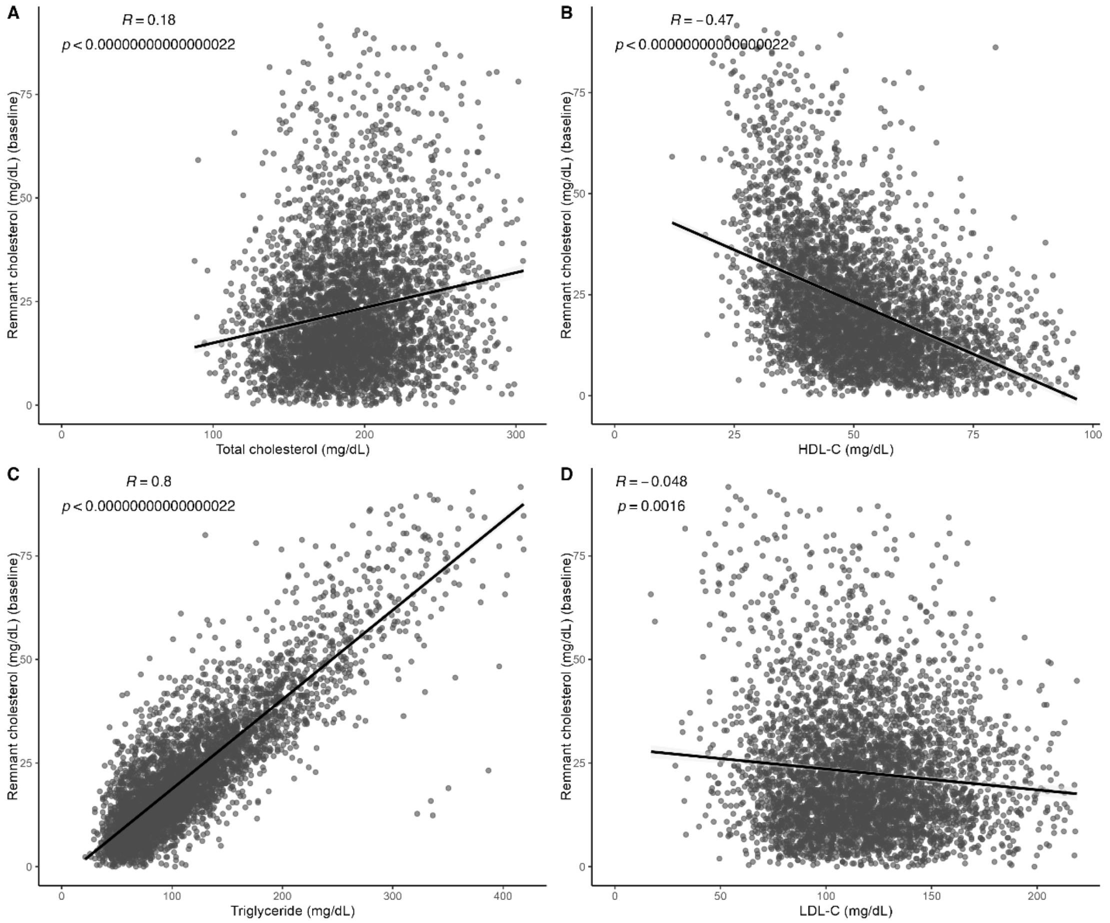

Abbreviations: TC: total cholesterol; TG: triglycerides; HDL-C: high-density lipoprotein cholesterol; LDL-C: low-density lipoprotein cholesterol.
